# Lower Superoxide Dismutase 2 (SOD2) Protein Content in Mononuclear Cells Is Associated with Better Survival in Patients with Hemodialysis Therapy

**DOI:** 10.1155/2016/7423249

**Published:** 2016-08-18

**Authors:** Katharina Krueger, Jianlin Shen, Alexandra Maier, Martin Tepel, Alexandra Scholze

**Affiliations:** ^1^Institute of Vegetative Physiology, Charité-Universitätsmedizin Berlin, 10117 Berlin, Germany; ^2^Department of Orthopedics, The Second Affiliated Hospital of Soochow University, Jiangsu, Suzhou 215006, China; ^3^Institute of Molecular Medicine, Cardiovascular and Renal Research, University of Southern Denmark, 5000 Odense C, Denmark; ^4^Department Nephrology, Charité-Universitätsmedizin Berlin, Campus Benjamin Franklin, 12203 Berlin, Germany; ^5^Department of Nephrology, Odense University Hospital, 5000 Odense C, Denmark; ^6^Institute of Clinical Research, University of Southern Denmark, 5000 Odense C, Denmark

## Abstract

Mitochondrial superoxide dismutase 2 (SOD2) converts superoxide anions to hydrogen peroxide and oxygen. Human data on SOD2 protein content in chronic kidney disease (CKD) are sparse and mortality data are lacking. We investigated SOD2 protein content in monocytes from patients with hemodialysis therapy (*n* = 81), CKD stage 1–5 (*n* = 120), and healthy controls (*n* = 13) using in-cell Western assays. SOD2 protein decreased from CKD stage 1 until stage 4 whereas it increased again in stage 5 with and without hemodialysis. SOD2 gene expression, analyzed by quantitative real-time PCR, was not significantly different between the groups. Elevating cellular superoxide production reduced SOD2 protein content. This effect was abolished by the superoxide dismutase mimetic Tempol. Using gelelectrophoresis and Western blot we did not detect nitrotyrosine modifications of SOD2 in CKD. Finally, in patients with CKD stage 5 with hemodialysis therapy higher than median SOD2 protein content was associated with higher all-cause mortality. In conclusion, SOD2 protein content declined in CKD until stage 4 while SOD2 gene expression did not. Increased cellular superoxide anion production might affect SOD2 protein content. In advanced CKD (stage 5) SOD2 protein content increased again, but higher than median SOD2 protein content in these patients did not confer a survival benefit.

## 1. Introduction

In chronic kidney disease (CKD) oxidative stress occurs frequently and has been proposed to be a central mechanism in the pathogenesis of CKD progression and CKD associated complications and mortality [1]. In particular dialysis patients show a higher mortality, and the cardiovascular mortality in patients with dialysis treatment is 10 to 20 times higher compared to the general population [2]. Oxidative stress describes the imbalance between formation of reactive oxygen species, like superoxide anions and hydrogen peroxides, and antioxidant defense systems [3]. Increased production of reactive oxygen species due to inflammation or decreased antioxidant capacity leads to lipid peroxidation and oxidation of proteins, carbohydrates, and amino acids [4]. Reactive oxygen species are normal by-products of cellular metabolism [5]. It has been estimated that 1 to 3% of the oxygen consumed by mitochondria is reduced to reactive oxygen species such as superoxide anion which is generated by one-electron-reduction of molecular oxygen [6, 7]. Superoxide dismutase (SOD) catalyzes the dismutation of superoxide anions to oxygen and hydrogen peroxide and thus represents a major antioxidant defense mechanism [8]. In mammals, different superoxide dismutase isoforms exist: superoxide dismutase 1 (SOD1, cytosolic copper-zinc superoxide dismutase), superoxide dismutase 2, and superoxide dismutase 3 (SOD3, extracellular copper-zinc superoxide dismutase) (for review see Fukai and Ushio-Fukai [9]). Superoxide dismutase 2 (SOD2, manganese superoxide dismutase) is one of the three isoforms and is located in mitochondria [10].

Early studies with mutant mouse models showed the importance of SOD2 in mitochondria [11, 12]. In the homozygous mutant mouse (Sod−/−) a complete loss of SOD2 protein results in impairment of further mitochondrial enzymes followed by death of animals in 1 to 20 days after birth. Heterozygous Sod2+/− mice have shown increased oxidative stress [12].

In chronic kidney disease, contrasting results about SOD2 enzyme were reported. While in a uremic rat model Lim et al. found reduced SOD2 protein content in the liver Finch et al. found increased SOD2 protein in the kidney [13, 14]. In human CKD SOD2 studies are so far limited to gene expression analyses in peripheral white blood cells. Akiyama et al. did not find a significant difference in SOD2 gene expression between healthy subjects and patients with CKD or CKD and hemodialysis treatment while Zaza et al. reported in 15 peritoneal dialysis patients a significantly higher SOD2 gene expression compared to healthy controls [15, 16]. SOD2 protein analyses in human CKD as well as mortality analyses in relation to SOD2 protein content are lacking.

We therefore investigated SOD2 protein content and gene expression in patients with CKD and analyzed survival rates in CKD stage 5 patients with hemodialysis therapy in relation to SOD2 protein.

## 2. Materials and Methods

A total of 81 consecutive patients with chronic kidney disease stage 5 undergoing maintenance hemodialysis, 120 patients with chronic kidney disease stages 1–5 without hemodialysis treatment, and 13 healthy control subjects were investigated. Written informed consent was obtained from each subject and ethical approval was given by the local ethics committee. Hemodialysis patients were dialyzed 4-5 hours three times per week using biocompatible membranes. Blood samples from hemodialysis patients were taken before the start of the hemodialysis session.

We investigated monocytes from peripheral blood as these cells are of special interest in CKD patients. The monocyte-macrophage lineage is involved in different pathogenic processes in CKD: systemic inflammation [17], vascular disease and atherosclerosis [18–21], kidney injury [22], and impaired immune function [23]. Monocytes were isolated from heparinized blood using superparamagnetic polystyrene beads coated with a primary monoclonal antibody specific for the CD14 membrane antigen (Invitrogen DYNAL, Norway) and washed several times in Hanks balanced salt solution (HBSS).

For protein analyses by Western blotting monocytes were lysed in homogenization buffer (containing 50 mmol/L Tris-HCl, pH 8; 100 mmol/L NaCl, 100 mmol/L *β*-mercaptoethanol, 50 mmol/L NaF, 2 mmol/L ethylenediaminetetraacetic acid, and complete mini protease inhibitor cocktail (Roche Diagnostics; Germany)); proteins were separated by 12.5% sodium-dodecyl-sulfate polyacrylamide gel electrophoresis (SDS-PAGE) at 150 V for 90 minutes and transferred to pure nitrocellulose membranes (Biorad; USA). Membranes were blocked with Odyssey blocking buffer (Licor biosciences; USA) and incubated with primary antibodies against superoxide dismutase 2 (SOD2) and beta-actin (Abcam). After washing with HBSS, the membranes were incubated with Alexa Fluor680-allophycocyanin-fluorescence-labelled (MoBiTec, USA) or IRDye800CW-infrared fluorescent dye-labelled (biomol, Germany) secondary antibodies. Imaging was performed using the Odyssey infrared imaging system (Licor biosciences; USA) at 700 nm or 800 nm emission with an excitation wavelength of 680 nm or 780 nm, respectively. Our experiments confirmed the specificity of the used SOD2 and beta-actin antibodies in human monocytes.

For the detection of nitrotyrosine the membranes were blocked with Odyssey blocking buffer (Licor biosciences; USA) and incubated with primary antibodies against nitrotyrosine (Chemicon, Millipore; USA). After washing with HBSS, the membranes were incubated with horseradish peroxidase-conjugated anti-rabbit secondary antibodies (DakoCytomation; Denmark). Visualization was performed using the ECL chemiluminescence system (Amersham, USA).

For the quantification of SOD2 protein content we performed in-cell Western assays of monocytes as recently described by our group [24]. The method provides a sum measurement for different posttranslationally modified and nonmodified forms of a protein (protein species) that can be detected with the primary antibody. We have proven this concept in a publication about superoxide dismutase 1 (SOD1) protein species, where we investigated SOD1 protein content in monocytes of CKD patients in parallel with two-dimensional gel electrophoresis. The latter revealed that our SOD1 sum analysis of protein content in in-cell Western assay covered at least 6 SOD1 protein species [25].

Human monocytes in 96 well plates were permeabilized with Triton X100 and coincubated with the primary antibodies against SOD2 and beta-actin (Abcam) for 2 hours. After washing steps, monocytes were coincubated with corresponding secondary antibodies (see below) for 1 hour. Imaging was performed at 700 nm or 800 nm emission with an excitation wavelength of 680 nm or 780 nm. The protein content of SOD2 was always normalized to the beta-actin protein content of the same cells. Control experiments were performed without incubation of primary antibodies.

For activation and inhibition experiments phorbol-myristate-acetate (PMA, Sigma-Aldrich, final concentration 100 ng/mL) and 4-hydroxy-2,2,6,6-tetramethylpiperidin-1-oxyl (Tempol, Sigma-Aldrich, final concentration 100 *μ*M) were used (incubation time 3 hours).

RNA isolation, transcription, and quantitative real-time PCR were performed according to manufacturer's descriptions (High Pure RNA Isolation Kit, Transcriptor First Strand cDNA Synthesis Kit, LightCycler®FastStart DNA MasterPlus SYBR Green I Kit; Roche; Germany). The following primers for real-time PCR were used:

 SOD2/NM_000636:
 F-5′- ggt ggt cat atc aat cat ag -3′;
 R-5′- agt gga ata agg ttt gtt gt -3′ (260 bp);
 Beta-actin/NM_001101:
 F-5′- aac tgc tta gca ccc ctg gc -3′;
 R-5′- atg acc ttg ccc aca gcc tt -3′ (200 bp);

The PCR conditions using a LightCycler 2.0 Instrument (Roche Diagnostics, Germany) were as follows: 95°C for 10 min and 40 cycles of 95°C for 10 s; 55°C (for SOD2) or 60°C (for beta-actin) for 10 s; and 72°C for 10 s. Normalized ratios of SOD2 mRNA expression were calculated relative to housekeeping gene beta-actin mRNA expression including efficiency correction and calibrator normalization. PCR products were also size-fractionated on 1.0% agarose gels and visualized by ethidium bromide staining.

### 2.1. Statistics

Data are given as median and interquartile range. Data between groups were compared using Mann-Whitney test or Kruskal-Wallis test and Dunn's multiple comparison posttest, as appropriate. Kaplan Meier survival curves were compared using log-rank test (GraphPad prism software, version 5.0, GraphPad Software, San Diego, CA). All statistical tests were two-sided. A two-sided value of *p* less than 0.05 was considered statistically significant.

## 3. Results

**Figure 1:**
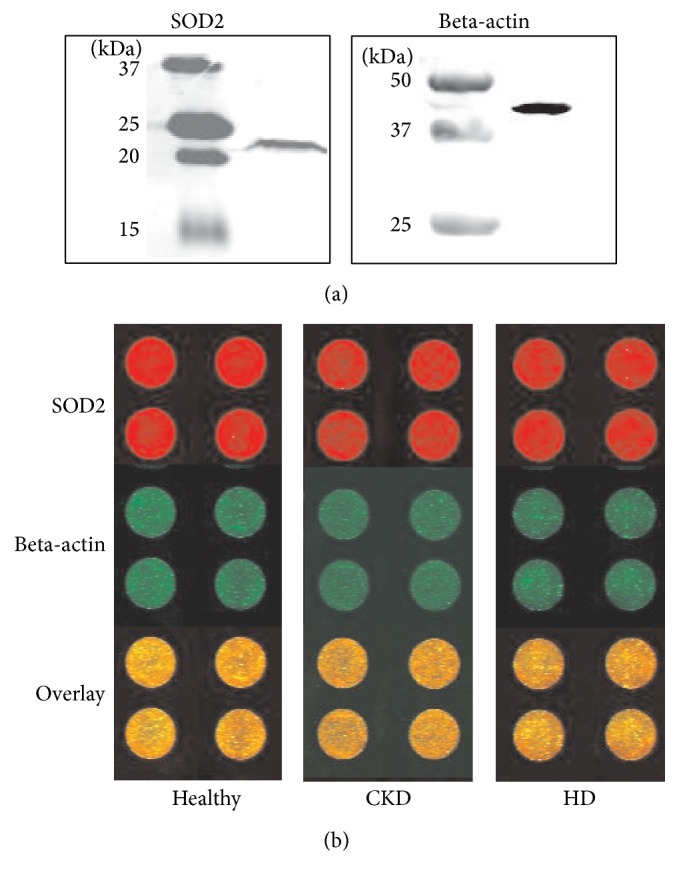
(a) Detection of proteins SOD2 and reference protein beta-actin in monocytes by immunoblotting. (b) Representative in-cell Western assay for quantification of SOD2 protein content in monocytes from a healthy control subject (Healthy), a patient with chronic kidney disease (CKD), and a hemodialysis patient (HD). Upper panels show SOD2 protein content (red fluorescence); middle panels of beta-actin (green fluorescence) and lower panels show overlay. Fluorescence intensities were analyzed in quadruplicate for each sample.

We investigated superoxide dismutase 2 (SOD2) protein in monocytes from patients with chronic kidney disease (CKD) and healthy controls using in-cell Western assays (subject characteristics given in Table 1). First, the specificity of the SOD2 and beta-actin antibodies used in our experiments was shown by Western blotting (Figure 1(a)). We demonstrated SOD2 and beta-actin protein detection in monocytes with molecular weights of 21 kDa and 41 kDa.  Figure 1(b) shows representative in-cell Western assays of SOD2 and beta-actin protein in monocytes from healthy control subject, a patient with CKD, and a hemodialysis patient (quadruplicate determination for each subject).

**Figure 2:**
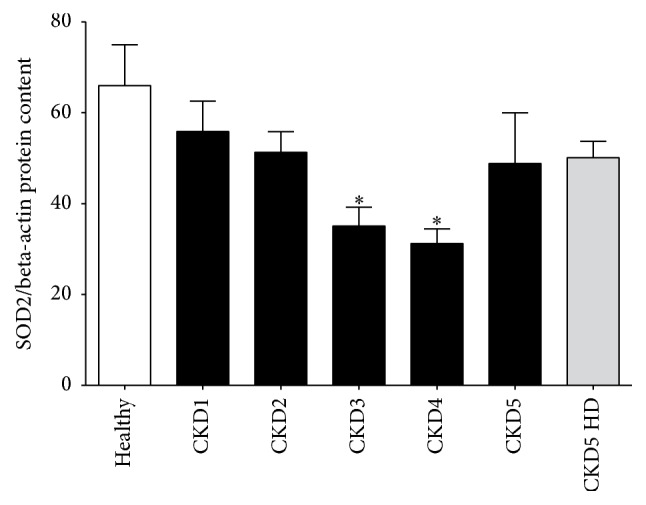
J-shaped pattern of SOD2 protein content. Quantification of SOD2 protein content relative to housekeeping protein beta-actin by in-cell Western assays. The figure shows the comparison between healthy control subjects, patients with CKD stage 1 through 5, and hemodialysis patients (CKD5 HD); ^*∗*^ *p* < 0.05 compared to healthy controls by Dunn's multiple comparison posttest.

Next, SOD2 protein content was analyzed for patients with all CKD stages, patients with hemodialysis therapy, and healthy controls.  Figure 2 shows the summary data for SOD2 protein content which is decreased with declining glomerular filtration rate until CKD stage 4. In contrast, patients with CKD stage 5 without and also with hemodialysis therapy then show again higher SOD2 protein content so that a J-shaped pattern results. Kruskal-Wallis analysis showed a significantly different distribution of SOD2 protein content between all groups (*p* < 0.002).

**Figure 3:**
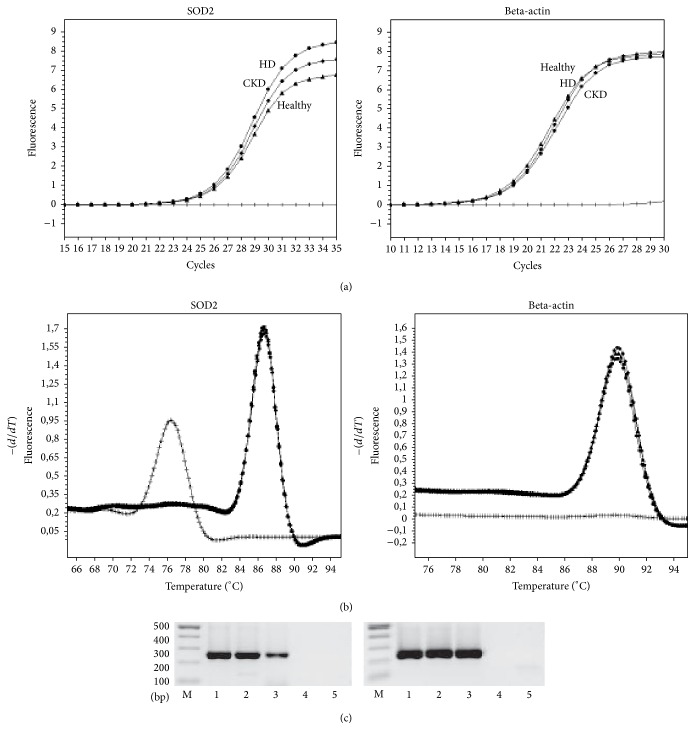
(a) Representative amplification curves of the quantitative real-time PCR for SOD2 and the housekeeping gene beta-actin in hemodialysis patients (HD, circles), patients with chronic kidney disease (CKD, squares), and control subjects (healthy; triangles). Curves with x-symbols indicate the no-template control in the PCR. (b) Melting analysis after amplification of SOD2 and the housekeeping gene beta-actin. The melting curve analysis confirmed the presence of one single peak in hemodialysis patients (HD, circles), patients with chronic kidney disease (CKD, squares), and healthy control subjects (healthy; triangles). Curves with x-symbols indicate the no-template control in the PCR and show a peak at lower temperature representing primer-dimers in the no-template reaction. (c) Example of size fractionation of PCR products on 1.0% agarose gels. M denotes the base pair DNA marker, lane 1 hemodialysis patient, lane 2 CKD patient, lane 3 healthy control, lane 4 negative control (no reverse transcriptase), and lane 5 negative control (no template). Left side shows PCR products for SOD2; right side shows PCR products for beta-actin.

**Figure 4:**
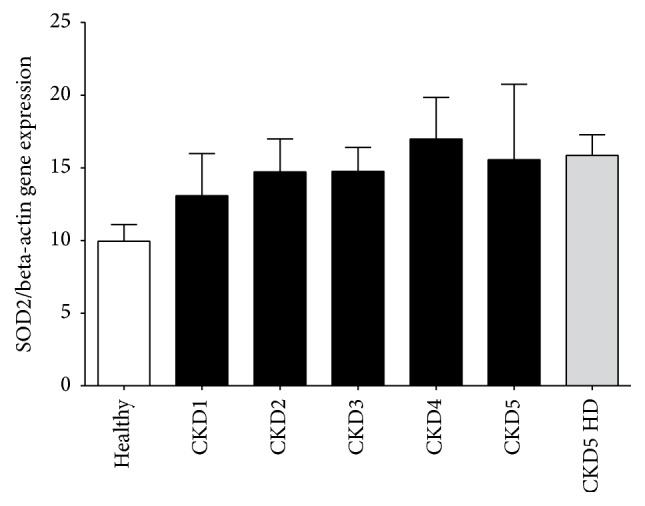
SOD2 mRNA expression analyzed by quantitative real-time PCR normalized to beta-actin in monocytes from healthy subjects (healthy), patients with chronic kidney disease (CKD1–5), and hemodialysis patients (CKD5 HD); *p* = 0.10 in the Kruskal-Wallis analysis.

In addition to quantification of the protein content, we investigated the SOD2 gene expression in monocytes from hemodialysis patients, patients with CKD, and healthy control subjects. Figures 3(a) and 3(b) present typical amplification and melting curves for SOD2 mRNA and the housekeeping gene beta-actin mRNA from a hemodialysis patient, a patient with CKD, and a control subject. PCR products were also size fractionated on 1.0% agarose gels and stained by ethidium bromide (Figure 3(c)). The SOD2 mRNA expression levels from patients with CKD and healthy control subjects are shown in Figure 4. The relative SOD2 to beta-actin gene expression in monocytes is expressed as ratio. Although there seemed to be a trend to increased SOD2 gene expression from healthy to CKD stage 4 patient the Kruskal-Wallis analysis did not show a significant difference in the distribution of SOD2 gene expression between the groups (*p* = 0.10).

**Figure 5:**
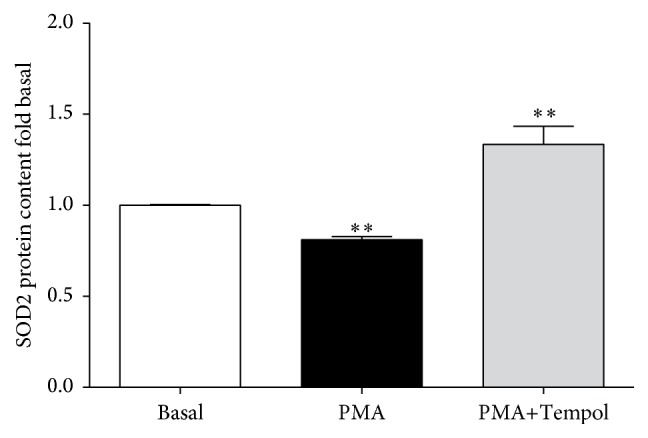
SOD2 protein content in cells under baseline conditions (control) and after incubation with PMA to stimulate cellular superoxide production with (*n* = 6) and without (*n* = 8) the SOD-mimetic Tempol. ^*∗∗*^ *p* < 0.01 versus basal.

Since we did not detect changes in SOD2 gene expression that explained the differences which we found in SOD2 protein content we investigated the effect of an increased cellular superoxide production on SOD2 protein content in mononuclear cells. To stimulate cellular superoxide production mononuclear cells were treated with PMA in the presence and absence of the SOD-mimetic Tempol.  Figure 5 shows changes in SOD2 protein after cell incubation with PMA with and without Tempol. The protein content of SOD2 in the cells at baseline (1.00 (0.98–1.02)) was reduced after incubation with PMA (0.82 (0.77–0.86); *n* = 8) whereas the addition of Tempol significantly increased SOD2 protein content (1.22 (1.02–1.44; *n* = 6); each *p* < 0.01).

We also performed SDS-PAGE and Western blot analyses to search for nitrotyrosine modifications on SOD2 proteins in healthy subjects (H) and CKD stage 4 (CKD) and CKD stage 5 HD (HD) patients. No protein staining at the expected site of SOD2 bands was detected by the anti-nitrotyrosine antibody (Supplementary Figure 1(C) in Supplementary Material available online at http://dx.doi.org/10.1155/2016/7423249).

**Figure 6:**
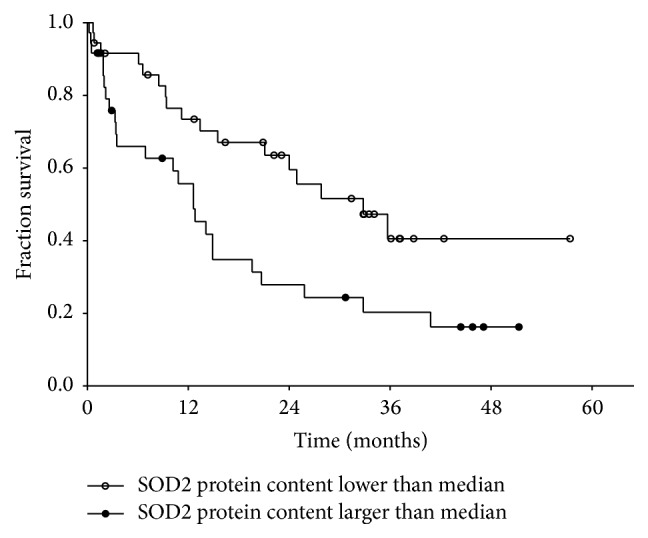
Kaplan Meier survival curves of hemodialysis (CKD5 HD; *n* = 81) patients according to the SOD2 protein content in peripheral blood monocytes (Chi square, 6.25; *p* < 0.05 by log-rank test).

Finally, we were interested in the relation between SOD2 protein content in monocytes of CKD stage 5 patients with hemodialysis therapy and all-cause mortality. We divided the patients in a group with SOD2 protein below and a group with SOD2 protein above the median (SOD2/beta actin protein = 42.54).  Table 2 shows a comparison of the clinical parameter of these two patient groups. As indicated in Figure 6 survival was significantly better in hemodialysis patients with lower SOD2 protein content in peripheral blood monocytes (Chi square, 6.25; *p* < 0.05 by log-rank test).

## 4. Discussion

We investigated SOD2 gene expression and protein content in monocytes of CKD patients. Also, in hemodialysis patients, a population with reportedly high all-cause and cardiovascular mortality, we analyzed the relation between SOD2 protein content and mortality. The major findings in our study are the following: (1) SOD2 protein content showed a J-curve pattern with significantly lower values compared to healthy controls and a progressive reduction until CKD stage 4 followed again by higher SOD2 protein content in CKD stage 5 and patients with hemodialysis treatment. (2) The SOD2 gene expression was not significantly different between the groups. (3) Higher than median SOD2 protein content was associated with higher mortality in patients with hemodialysis therapy.

Both, the results on protein content and gene expression of SOD2 in our study, are interesting. In an earlier study our group investigated gene expression and protein content of the cytosolic superoxide dismutase isoform (SOD1) in monocytes of patients with CKD. In contrast to SOD2, the SOD1 protein was reduced in CKD showing the lowest protein content in patients with hemodialysis therapy; and SOD1 gene expression was increased in CKD, with a high significance in patients with hemodialysis [25]. These results are supported by the literature. In peripheral blood mononuclear cells of hemodialysis patients a significant increase of SOD1 mRNA compared to healthy controls was reported while SOD2 mRNA did not differ significantly from control [15]. The different gene expression of SODs in CKD could be explained by the multiple differences in gene regulation between SOD1 and SOD2 (for review see Miao and St. Clair [26]). With respect to the reduced SOD2 protein content found in CKD stages 3 and 4 in our present study different explanations are suggested by the literature. Two factors in CKD can contribute to an enhanced degradation of proteins: oxidative stress and uremia. Oxidative modification of SOD2 proteins has been described. Our own Western blot analyses did not suggest tyrosine nitration of SOD2 in CKD although refined analysis requires mass-spectrometric analyses of SOD2 protein from CKD patients and healthy subjects. While some authors using recombinant SOD2 protein showed tyrosine nitration and tyrosine oxidation upon treatment with peroxynitrite [27], protein biochemical analyses of SOD2 protein species from medulloblastoma cells showed tryptophan oxidation and histidine oxidations but no tyrosine nitration [28]. Therefore, it will be necessary to analyze oxidative SOD2 modifications for each pathogenic condition individually. Further insights could be revealed by adjacent analyses of SOD2 protein degradation and SOD2 enzymatic activity.

Our own results in the current study point to an involvement of reactive oxygen species in the regulation of SOD2 protein content since we observed a PMA-induced decrease of SOD2 protein in mononuclear cells that could be abolished by treatment with the SOD-mimetic Tempol. Furthermore, increased SOD2 protein degradation in the uremic environment may be involved. SOD2 protein was shown to be degraded by the ubiquitin-proteasomal pathway [29]. In CKD protein degradation via the ubiquitin-proteasomal pathway is enhanced [30, 31]. Generally, posttranslational protein modifications known for SOD2 from other cell types should also be considered for monocytes. These are tyrosine nitration and tyrosine oxidation [27], histidine and tryptophan oxidation [28], acetylation (for review see Zou et al. [32]), and ubiquitination [29].

Numerous studies have shown that the correlation between gene expression, quantified by messenger RNA (mRNA) analysis, and quantification of protein content is often limited (for review see [33, 34]). Specifically, for changes in protein content but unaltered mRNA levels a regulation of translation or regulatory changes of protein degradation can be underlying mechanisms.

The following conditions for instance have been described: reduced mRNA levels but constant protein content [35], constant mRNA levels but reduced protein content due to increased protein degradation [36], and constant mRNA levels but increased protein content due to decreased protein degradation [37]. Furthermore, our own group showed for superoxide dismutase 1 in monocytes from chronic kidney disease patients an increased mRNA level together with reduced superoxide dismutase 1 protein content [25].

The mechanisms underlying the increase of SOD2 protein in advanced CKD (CKD5 with and without hemodialysis) compared to CKD 3 and 4 that we observed and that resulted in a J-shape of SOD2 protein content are not known. In this respect it is of interest that an upregulation of SOD2 protein that was reported in uremic rats could be reversed by vitamin D receptor agonists [14]. Since advanced uremia is a state of 1,25-dihydroxyvitamin D deficiency but also of vitamin D receptor deficiency and dysfunction, vitamin D receptor-related uremic effects could be involved in the J-shape of SOD2 protein that we observed [38].

Finally, we observed a better survival in hemodialysis patients with a SOD2 protein content below the median of this patient group compared to those with SOD above the median. The underlying mechanism for this observation is not known but considering the above discussed connection to vitamin D metabolism an association of higher content of SOD2 protein with more severe uremic disturbance of vitamin D receptor signal transduction could be involved.

In future experiments it will be necessary to identify underlying SOD2 protein modifications using 2 DE, mass-spectrometry and immunoblotting.

Taken together, our study shows significant changes of SOD2 protein content with increasing degree of renal function impairment. We also proof, in line with our own previous research but also with many other groups, that in clinical-experimental research a parallel investigation of gene expression and protein content is indispensable. And finally, we provide the first report of SOD2 protein content together with its association with survival in CKD patients with hemodialysis therapy.

## Supplementary Material

Supplementary figure 1 shows analyses of SOD2 proteins by SDS-PAGE and Western blot. We show analyses of SOD2 protein content in healthy subjects, CKD stage 4 and CKD stage 5 HD patients (A), a comparison of protein motility of SOD2 proteins between healthy subjects, CKD stage 4 and CKD stage 5 HD patients (B), and a staining of monocyte cell lysates from healthy subjects, CKD stage 4 and CKD stage 5 HD patients with an anti-nitrotyrosine antibody (C).

## Figures and Tables

**Table 1:** Population characteristics of subjects for superoxide dismutase 2 protein comparisons. Values are given as median (interquartile range) or number (percent).

	*n*	Age (years)	BMI (kg/m^2^)	Gender male (%)
Healthy controls	13	46 (41–52)	23.6 (20.6–25.2)	7 (54)
CKD stage 1	32	56 (44–67)	24.6 (22.7–28.5)	23 (72)
CKD stage 2	22	65 (57–70)	27.5 (22.7–32.3)	12 (55)
CKD stage 3	28	71 (62–80)	26.1 (22.0–28.4)	18 (64)
CKD stage 4	29	67 (60–74)	26.7 (22.6–31.0)	22 (76)
CKD stage 5	9	71 (56–75)	26.2 (24.6–30.7)	3 (33)
CKD stage 5 HD^*∗*^	81	66 (57–73)	24.4 (22.2–28.5)	47 (58)

^*∗*^HD = hemodialysis therapy.

**Table 2:** Clinical and biochemical characteristics of CKD patients with hemodialysis therapy. Values are median (interquartile range).

Characteristics	Lower than median SOD2 protein	Higher than median SOD2 protein
Age (years)	66 (58–73)	66 (56–74)
Gender (male/female)	24/16	22/18
Body mass index (kg/m^2^)	24.4 (22.7–28.6)	24.3 (22.1–27.8)
Time since initiation of dialysis treatment (days)	244 (30–481)	295 (39–1193)
Hemoglobin (g/dL)	10.1 (9.3–11.4)	10.1 (8.6–12.0)
C-reactive protein (mg/dL)	2.7 (0.1–6.2)	2.6 (0.9–6.9)
