# Supplementary material for: Lower Superoxide Dismutase 2 (SOD2) Protein Content in Mononuclear Cells Is Associated with Better Survival in Patients with Hemodialysis Therapy
Source: Oxid Med Cell Longev. 2016 Aug 18;2016:7423249. doi: 10.1155/2016/7423249 (PMC5007362; doi:10.1155/2016/7423249)
Supplement: Supplementary file 1 — Supplementary figure 1 shows analyses of SOD2 proteins by SDS-PAGE and Western blot. We show analyses of SOD2 protein content in healthy subjects, CKD stage 4 and CKD stage 5 HD patients (A), a comparison of protein motility of SOD2 proteins between healthy subjects, CKD stage 4 and CKD stage 5 HD patients (B), and a staining of monocyte cell lysates from healthy subjects, CKD stage 4 and CKD stage 5 HD patients with an anti-nitrotyrosine antibody (C). [file 7423249.f1.pdf]

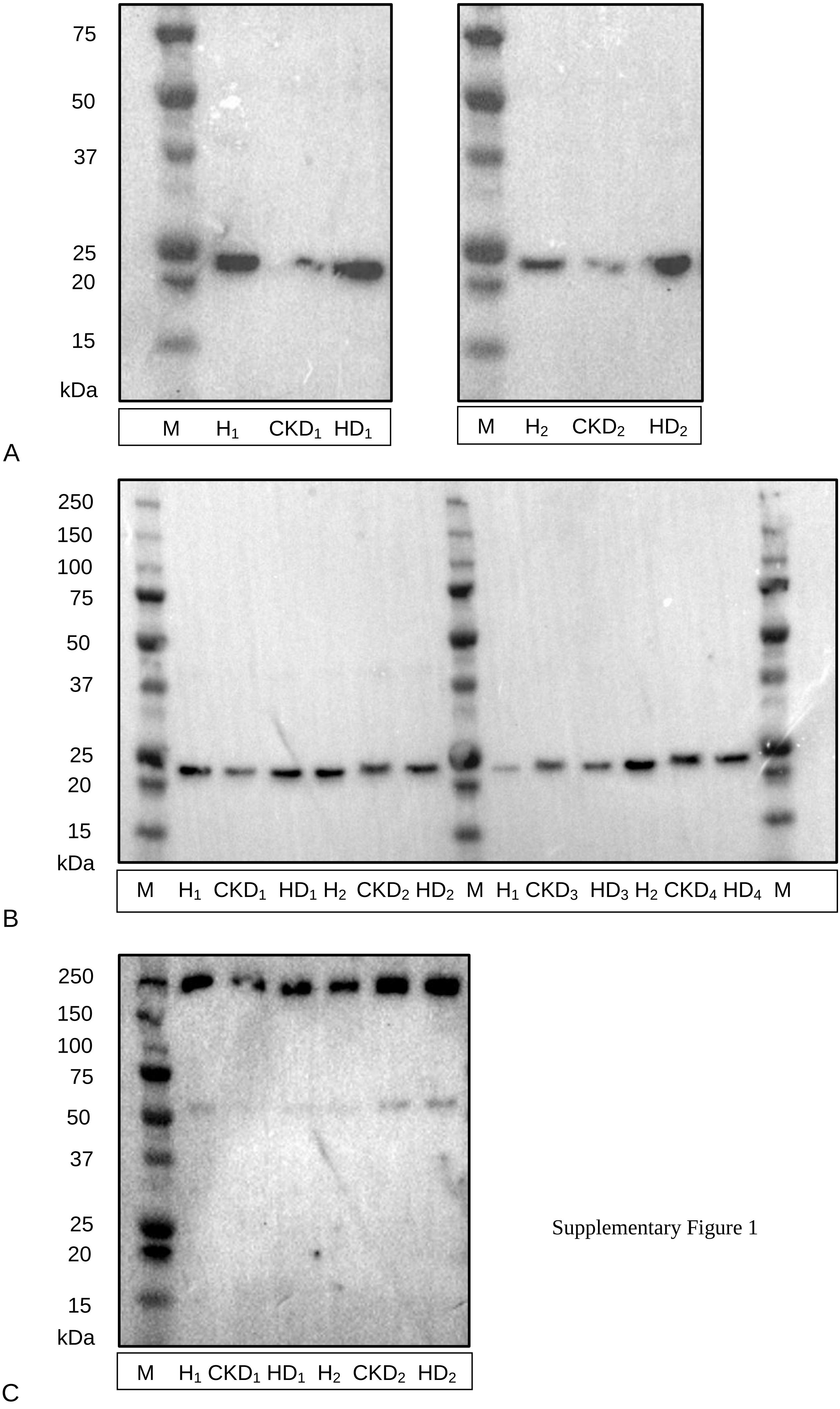

Supplementary Figure 1

Figure legend

Supplementary figure 1

A. Analyses of SOD2 protein content by SDS-PAGE gel and immunoblotting (M = marker). For the gel analyses we loaded each lane with an equal amount of monocyte lysate protein (10µg). In accordance with our results from the SOD2 protein quantification by in-cell Western assays SOD2 protein content was considerably lower in CKD stage 4 patients. Equal protein loading to the gel lanes therefore did not allow the intended comparison of SOD2 protein motility between different lanes.

B. Analyses of SOD2 protein motility by SDS-PAGE gel and immunoblotting (M = marker). We performed this analysis to compare SOD2 protein motility between healthy subjects, CKD stage 4 and CKD stage 5 HD patients. Compared to the gel analyses shown under A. we adjusted the protein loading to the different lanes to obtain a comparable amount of SOD2 protein per lane. The figure shows SOD2 proteins from CKD stage 4 (CKD) and CKD stage 5 HD (HD) patients in comparison to SOD2 from healthy subjects (H). There might be a slight change in the SDS-PAGE gel motility of SOD2 in CKD patients number 2 and 4 and HD patients number 2 and 4. For further differentiation of possible SOD2 protein species 2 DE and mass-spectrometric analyses of SOD2 protein from respective patients and healthy subjects are necessary.

C. Analyses of nitrotyrosine content in monocyte cell lysates by SDS-PAGE gel and immunoblotting (M = marker).

We performed this analysis to search for nitrotyrosine modifications on SOD2 proteins in healthy subjects (H), CKD stage 4 (CKD) and CKD stage 5 HD (HD) patients. No protein staining at the expected site of SOD2 bands was detected by the anti-nitrotyrosine antibody. Instead, a pronounced staining of a high molecular weight band is present that is not detected by the anti-SOD2 antibody (compare under B). Our immunoblot analysis does not suggest

tyrosine nitration of SOD2 in CKD but refined analysis requires mass-spectrometric analyses of SOD2 protein from respective patients and healthy subjects.
